# Chronic Cigarette Smoke Causes Oxidative Damage and Apoptosis to Retinal Pigmented Epithelial Cells in Mice

**DOI:** 10.1371/journal.pone.0003119

**Published:** 2008-09-01

**Authors:** Masashi Fujihara, Norihiro Nagai, Thomas E. Sussan, Shyam Biswal, James T. Handa

**Affiliations:** 1 Wilmer Eye Institute, Johns Hopkins School of Medicine, Baltimore, Maryland, United States of America; 2 Department of Environmental Health Sciences, Bloomberg School of Public Health, Johns Hopkins University, Baltimore, Maryland, United States of America; Ordway Research Institute, United States of America

## Abstract

The purpose of this study was to determine whether mice exposed to chronic cigarette smoke develop features of early age-related macular degeneration (AMD). Two month old C57Bl6 mice were exposed to either filtered air or cigarette smoke in a smoking chamber for 5 h/day, 5 days/week for 6 months. Eyes were fixed in 2.5% glutaraldehyde/2% paraformaldehyde and examined for ultrastructural changes by transmission electron microscopy. The contralateral eye was fixed in 2% paraformaldehyde and examined for oxidative injury to the retinal pigmented epithelium (RPE) by 8-oxo-7,8-dihydro-2′-deoxyguanosine (8-OHdG) immunolabeling and apoptosis by TUNEL labeling. Mice exposed to cigarette smoke had immunolabeling for 8-OHdG in 85±3.7% of RPE cells counted compared to 9.5±3.9% in controls (p<0.00001). Bruch membrane was thicker in mice exposed to smoke (1086±332 nm) than those raised in air (543±132 nm; p = 0.0069). The two most pronounced ultrastructural changes (severity grading scale from 0–3) seen were a loss of basal infoldings (mean difference in grade = 1.98; p<0.0001), and an increase in intracellular vacuoles (mean difference in grade = 1.7; p<0.0001). Ultrastructural changes to Bruch membrane in cigarette-smoke exposed mice were smaller in magnitude but consistently demonstrated significantly higher grade injury in cigarette-exposed mice, including basal laminar deposits (mean difference in grade = 0.54; p<0.0001), increased outer collagenous layer deposits (mean difference in grade = 0.59; p = 0.002), and increased basal laminar deposit continuity (mean difference in grade = 0.4; p<0.0001). TUNEL assay showed a higher percentage of apoptotic RPE from mice exposed to cigarette smoke (average 8.0±1.1%) than room air (average 0±0%; p = 0.043). Mice exposed to chronic cigarette smoke develop evidence of oxidative damage with ultrastructural degeneration to the RPE and Bruch membrane, and RPE cell apoptosis. This model could be useful for studying the mechanism of smoke induced changes during early AMD.

## Introduction

Age-Related Macular Degeneration (AMD) is the leading cause of blindness among the elderly in the United States. Due to the aging population, the number of people with advanced AMD will increase from 1.75 million now, to 3 million by 2020 [1]. The impact to the individual and the general public is devastating. In a value-based medical analysis, the deleterious effect of AMD on quality of life is markedly underestimated by both physicians and the public [2]. For example, the decrease in quantifiable quality of life from early AMD is equivalent to a patient with symptomatic human immunodeficiency virus infection or moderate cardiac angina. Currently, there is no effective preventive or treatment for early, non-neovascular AMD.

Oxidative stress has long been hypothesized to play a substantive role in the development of AMD due to the high oxidative stress environment of the fundus. The Age Related Eye Disease (ARED) study showed that high dose antioxidant vitamin therapy reduced the advancement of intermediate non-neovascular AMD, and that this benefit was associated with a reduction in plasma glutathione and cysteine oxidation [3]. While the genetic variations of several complement factors have been associated with AMD susceptibility, different studies also have identified a susceptibility locus for AMD may be located in or near the hypothetical LOC387715 gene [4], [5]. Kanda et al have confirmed that this locus was the susceptibility locus for AMD, and that this gene encodes a mitochondrial protein [6]. Interestingly, this locus may be associated with smoking, and the combination of the LOC387715 polymorphism and smoking confers a higher risk for AMD than either factor alone [7]. This finding, along with its identification as a mitochondrial protein, raises suspicion for a role of the oxidative defense response in this disease. Further evidence for genetic susceptibility related to oxidative stress has been provided by Canter et al, who have correlated the mitochondrial DNA polymorphism A4917G with AMD [8] and Kimura et al, who showed that a polymorphism in superoxide dismutase 2 (SOD2) is associated with AMD in a small subset of patients [9]. Cigarette smoke, which can be considered a strong chemical oxidant, has the strongest epidemiological link with AMD [10]. However, experimental evidence is lacking for injury to the retinal pigmented epithelium (RPE), a principal cell type involved in AMD. Critical host factors that protect the RPE from oxidative injury may determine its susceptibility to tissue destruction or modify the intensity of inflammatory reaction associated with AMD.

RPE cell apoptosis and basal deposits, or accumulations of heterogeneous debris in Bruch membrane (BrM), are two critical histopathologic changes that are well recognized to occur during the development of early AMD [11]–[24]. We used these established changes as endpoints for a study designed to determine if cigarette smoke induces evidence of changes associated with AMD. Mice were exposed to 6 months of cigarette smoke in a chamber that produces emphysema with evidence of oxidative damage [25]. In this manuscript, we explored whether mice exposed to cigarette smoke developed these two cardinal features of early AMD using this protocol.

## Results

### Evidence of Increased Oxidative Stress in the RPE of Mice Exposed to Cigarette Smoke for 6 Months

**Figure 1:**
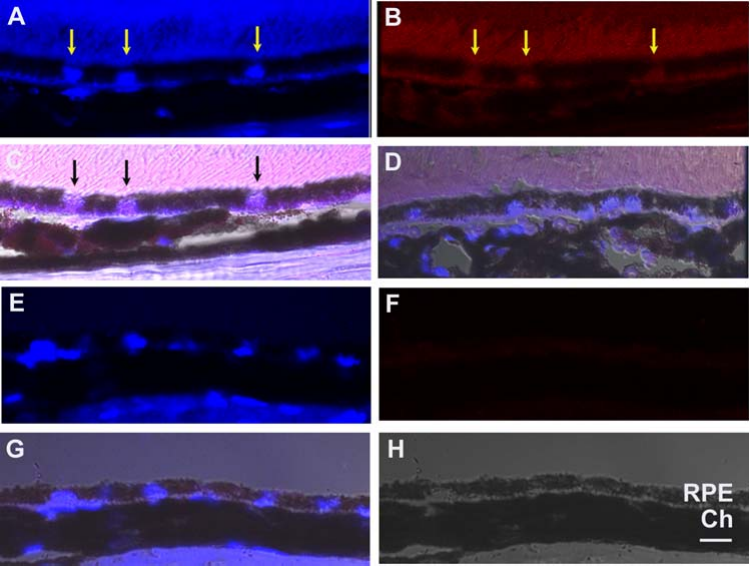
Immunohistochemistry of 8-OHdG nuclear labeling of the RPE. 8 month C57Bl6 mouse exposed to smoke for 6 months showing A. DAPI labeled nuclei (arrows); B. 8-OHdG labeled RPE nuclei (arrows); C. Merged image of A and B with the Brightfield image showing violet nuclei (arrows); D. Merged image of DAPI and IgG1 control image with Brightfield image overlay. 8 month old C57Bl6 mouse raised in air showing DAPI labeled RPE nuclei in E and 8-OHdG immunostaining in F; G. Merged DAPI and 8-OHdG immunostained image with Brightfield image overlay showing blue nuclei in H. Brightfield image. RPE, retinal pigmented epithelium; Ch, choroid. Bar = 15 μm. Figure shows representative images from N = 10 mice (50 samples/mouse).

Immunohistochemical staining with an anti-8OHdG antibody was used to assess evidence of oxidative damage in the RPE of mice exposed to air or cigarette smoke. This antibody is highly specific for 8-OHdG, and our laboratory has previously used this antibody to identify oxidative DNA damage [26], [27].We quantified the nuclei that were immunostained per 100 RPE cell nuclei. In mice housed in filtered room air (n = 5), 9.5±3.9% of RPE cell nuclei exhibited staining for 8-OHdG, whereas significantly more (85±3.7%; p<0.00001) stained nuclei were observed in mice exposed to cigarette smoke (n = 5; Figure 1).

### Ultrastructural Injury to the RPE and Bruch Membrane in Mice Exposed to Cigarette Smoke

**Figure 2:**
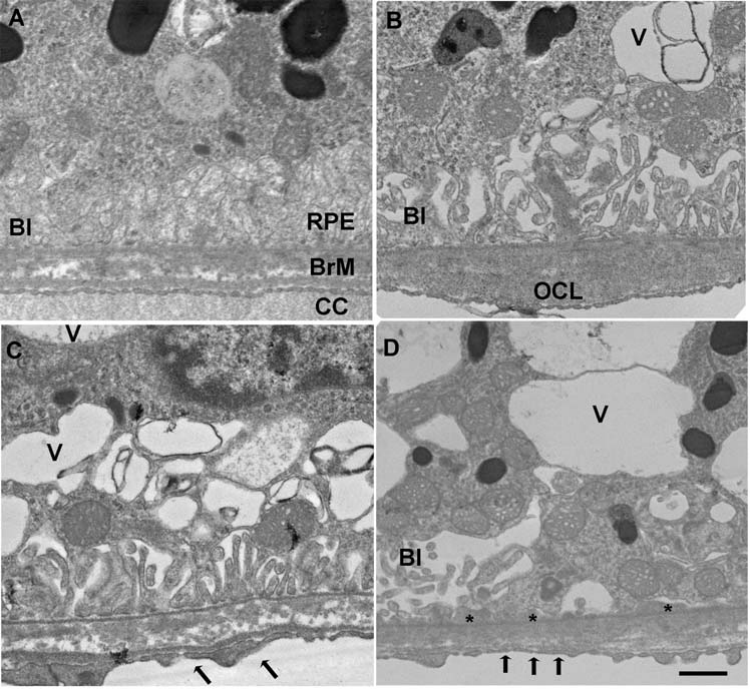
Transmission electron microscopy of the RPE-Bruch membrane-choriocapillaris of mice exposed to air (A) or cigarette smoke (B-D). A. The RPE has normal cytoplasm and regular basal infoldings (BI) Bruch membrane (BrM) is unthickened and without deposits. The choriocapillaris (CC) has regular fenestrations. B. A membranous vacuole (V) appears in the cytoplasm and the basal infoldings (BI) are fewer and dilated in the RPE. A small outer collagenous layer (OCL) deposit is seen in Bruch membrane. C-D. More severe membranous vacuoles appear in the cytoplasm than in B. The basal infoldings are fewer and dilated. The choriocapillaris fenestrations are fewer (arrows). D. The RPE has multiple, large vacuoles in the cytoplasm and the basal infoldings are fewer and dilated, or absent. Thin basal laminar deposits (*) are seen where the basal infoldings are absent. Bar = 500 nm.

The RPE of 8 mo old mice raised in air appeared healthy with normal basolateral infoldings (Figure 2A). Bruch membrane maintained a pentalaminar structure composed of the RPE basement membrane, inner collagenous layer, middle elastic layer, outer collagenous layer, and basement membrane. The choriocapillaris endothelium appeared healthy with fenestrations. We chose RPE basolateral infoldings and cytoplasmic vacuoles as indicators of RPE cell degeneration because loss of basal infoldings is a marker of epithelial injury [28]–[30] and cytoplasmic vacuoles have been identified in RPE that overlie drusen deposits [13]. Figure 2 also shows an 8 mo old mouse that has been exposed to chronic cigarette smoke exhibiting ultrastructural injury to the RPE-Bruch membrane. The RPE basolateral infoldings are dilated and fewer in number, and contain large cytoplasmic vacuoles. Bruch membrane shows an outer collagenous layer deposit while the choriocapillaris has focal loss of fenestrations.

**Figure 3:**
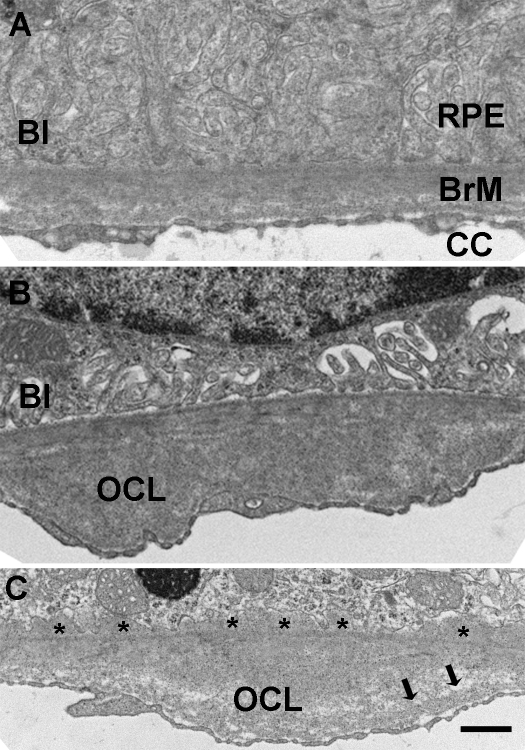
Transmission electron microscopy of Bruch membrane of mice exposed to air (A) or cigarette smoke (B,C). A. Mouse exposed to air shows regular basal infoldings (BI) of the RPE and unthickened Bruch membrane (BrM). B. The RPE show loss of basal infoldings. Bruch membrane is thickened due to an outer collagenous layer deposit (OCL). C. The RPE show more severe loss of basal infoldings than in (B). Bruch membrane contains early basal laminar deposits (*), OCL, and choriocapillaris (CC) basement membrane reduplication (arrows). Bar = 500 nm.

Bruch membrane thickens with aging. We therefore measured Bruch membrane thickness, and found that Bruch membrane was thicker in mice exposed to smoke (n = 10, 1086±332 nm) than those raised in air (n = 10, 543±132 nm; p = 0.0069). Part of this thickness is due to outer collagenous layer deposits and basal laminar deposits, as shown in Figure 3.

The severity of ultrastructural changes to the RPE-Bruch membrane-choroid was rated using a scale of 0 (no change) to 3 (most severe changes)according to our previously published protocol based on Cousins et al, and semi-quantified using regression analysis [31]. For each ultrastructural outcome considered, cigarette-exposed mice (n = 10) demonstrated significantly higher severity grades, consistent with more severe injury than mice raised in air (n = 10). By regression analysis, the two most pronounced changes were a loss of basolateral infoldings (mean difference in grade = 1.98; p<0.0001), and an increase in intracellular vacuoles (mean difference in grade = 1.7; p<0.0001). Other ultrastructural changes were smaller in magnitude, but consistently demonstrated significantly higher grade injury in cigarette-exposed mice. For example, cigarette-exposed mice had greater thickness and change in heterogeneous content of basal laminar deposits (mean difference in grade = 0.54; p<0.0001), increased outer collagenous layer deposits (mean difference in grade = 0.59; p = 0.002), and increased continuity (mean difference in grade = 0.4; p<0.0001). The choriocapillaris basement membrane was thicker in mice exposed to smoke than those reared in room air. Finally, the choriocapillaris endothelium showed mild loss of fenestrations with smoke exposure (mean difference in grade = 0.52; p<0.0001).

### RPE Apoptosis is Increased in Mice Exposed to Cigarette Smoke

**Figure 4:**
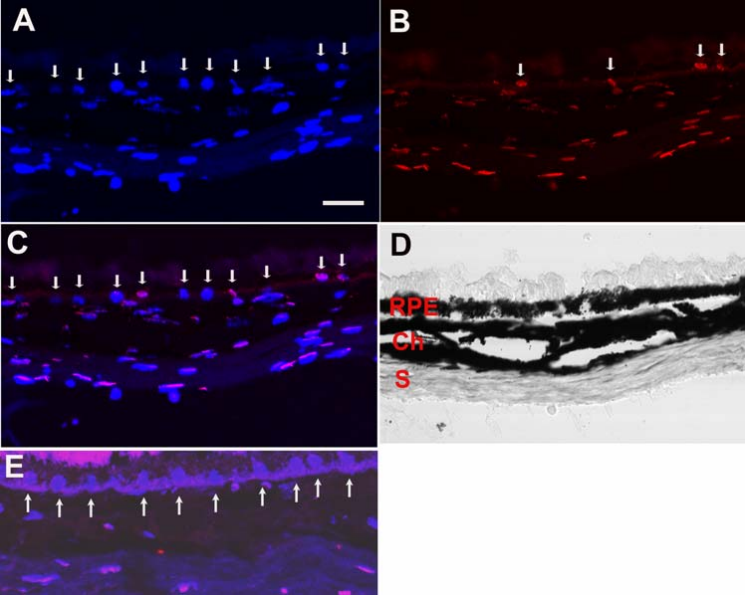
TUNEL labeling of RPE cells from mice exposed to cigarette smoke for 6 months. A. TUNEL labeled (red) RPE nuclei are indicated by the arrows. B. Nuclei are stained with DAPI (blue), as labeled by the arrows. C. Merged image of A and B separating TUNEL from DAPI only stained nuclei. D. Brightfield image of the RPE, choroid (Ch) and sclera (S). E. Merged image of a mouse raised in air for 6 months. Arrows point to blue DAPI without red TUNEL labeling. Bar = 15 μm.

To determine whether chronic exposure to cigarette smoke induced apoptosis of RPE cells in vivo, we conducted TUNEL staining on the RPE-choroid of mice exposed to cigarette smoke and room air. Figure 4 shows TUNEL-positive RPE cells from a mouse exposed to cigarette smoke. TUNEL showed a higher percentage of apoptotic RPE from mice exposed to cigarette smoke (n = 5, average 8.0±1.1%) than room air (n = 5, average 0.0±0%; p = 0.043).

## Discussion

In this study, we observed injury to the RPE and Bruch membrane of mice after chronic exposure to cigarette smoke. The RPE had specific ultrastructural changes that are associated with injury that have been observed in AMD. RPE apoptosis is an established change in aging, early AMD, and later stages of AMD such as geographic atrophy [11], [12]. The results from this study suggest that cigarette smoke plays a role in RPE changes associated with AMD. Increased oxidative damage, as assessed by DNA adduct formation, were measured in the RPE of mice exposed to cigarette smoke with immunostaining for 8-OHdG. These results implicate a role for oxidative damage to the RPE during this process. We also identified Bruch membrane thickening and mild basal deposits. Basal deposits and Bruch membrane thickening are hallmark changes of aging. The mild severity of basal deposits falls short of changes associated with AMD. Cigarette smoke had a greater impact on the RPE than Bruch membrane, which suggests that multiple factors are involved in the development of the full blown AMD phenotype.

Cigarette smoke is the strongest environmental risk factor associated with AMD. The epidemiologic data, such as the Beaver Dam Eye Study, suggest that smoking induces early AMD as well as progression of AMD, compared to nonsmokers [32]. Clemons et al in the ARED study group also found that cigarette smoking was associated with progression from early to advanced AMD [33]. Khan et al showed that pack-year smoking is strongly correlated with AMD while smoking cessation reduces the risk of developing AMD [34]. The RPE appears to be a specific target of cigarette smoke associated changes. Mitchell et al in the Blue Mountains Eye Study, showed that cigarette smoke is associated with increased risk of RPE abnormalities [35]. Likewise, in the AREDS cohort, cigarette smoking was associated with development of geographic atrophy, which is characterized by atrophy of the RPE, and cell death from apoptosis.

Cigarette smoke is a strong oxidant generated by 4700 chemical components [25]. The 8-OHdG immunohistochemical experiments indicate that despite a significant anti-oxidant defense system, the RPE developed oxidative DNA damage in mice exposed to cigarette smoke. The most obvious ultrastructural sign of injury to the RPE was enlargement and loss of basolateral infoldings, which is an established marker of epithelial cell injury from a number of etiologies including oxidative stress [28]–[30]. We used vacuole formation as a second sign of RPE change because it is known to occur in RPE cells overlying drusen deposits [13]. We presume that the degree of oxygen free radicals generated from cigarette smoke was involved in ultrastructural damage to the RPE. Our TUNEL experiments showed a clear increase in RPE cell apoptosis in mice exposed to cigarette smoke. While there can be multiple stimuli for apoptosis of the RPE, the most likely stimulus in this model, is oxidative stress from cigarette smoke.

Previous models of oxidative stress have shown damage to the RPE. Gottsch et al used a mouse model of protoporphyria and exposure to blue light to induce choriocapillaris injury and sub-RPE basal laminar-like deposits that simulated aging-associated changes prior to the onset of AMD [36]. Hahn et al, using a ceruloplasmin and hephaestin double knockout mouse that accumulates iron in the retina and RPE, developed an age-dependent retinal pigmented epithelium hypertrophy, hyperplasia, and death through enhanced oxidative stress [37]. Imamura et al showed in an SOD1 deficient mouse, oxidative damage to the RPE as well as development of drusen and thickened Bruch membrane [38]. In fact, Ferrington et al showed that RPE cell survival to an oxidant is tied to its ability to detoxify reactive oxygen species [39]. However, our lab and Lu et al have shown that the type of oxidant can result in different responses by the RPE [40], [41]. For example, Lu et al found that the antioxidant defense system of RPE cells protects well against damage to mitochondria and endoplasmic reticulum, but the cell is less able to handle oxidative damage at the cell surface. These results could explain the different phenotypes identified in the different models of oxidative stress. Since cigarette smoke is a complex chemical oxidant with an established epidemiologic link to AMD, it seems logical to use this model in determining the response from the RPE from oxidants in cigarette smoke.

Espinosa-Heidmann et al found that a shorter duration, higher concentration of cigarette smoke in 16 month old C57Bl6 mice induced ultrastructural changes to Bruch membrane and the choriocapillaris endothelium that are compatible with early AMD [42]. In their study, mice were exposed to more severe levels of cigarette smoke over a shorter period ( i.e. 2 hours per day, 5 days/week over 3.5 months), than the chronic experimental conditions that we selected (5 hours/day, 5 days/week over 6 months). The Espinosa-Heidmann et al protocol had higher levels of total suspended particulate (250 mg/m^3^) and carbon monoxide (600–750 ppm) compared to our levels of 90 mg/m^3^ and 350 ppm, respectively. In addition, they used significantly older mice than in our study. We selected our protocol based on evidence that this model induces emphysema in mice, and that AMD lesions are thought to develop over a long period of time [25]. The younger age allows us to isolate the effect of cigarette smoke on the RPE from the complex factors related to chronological aging, which remains the most common risk factor for AMD. It is difficult to determine what factors caused preferential injury to the RPE over Bruch membrane in our study. Interestingly, Espinosa-Heidmann et al did not find compelling ultrastructural evidence of RPE cell injury. However, they did not specifically study apoptosis. Given their more acute exposure of higher concentrations of cigarette smoke, it is possible that cells could die before showing ultrastructural evidence of injury. Alternatively, because of the significant anti-oxidant capability of the RPE, a chronic exposure to the oxidants in cigarette smoke might be necessary to cause RPE injury and apoptosis.

While this study provides data in support of a role for chronic cigarette smoke in RPE cell injury and apoptosis as it related to AMD, further work is necessary to provide a causal link. It is clear that other factors such as genetic susceptibility, the role of lipids, and chronic inflammation are important factors in the development of AMD. The value of this model is that each of these factors can be studied to determine whether cigarette smoking has a synergistic effect on important endpoints of AMD.

## Materials and Methods

### Animals and Care

An equal number of male and female C57Bl6 mice were fed standard rodent chow and water ad libitum, and kept in a 12-hour light-dark cycle. All experiments were conducted according to the ARVO Statement for the Use of Animals in Ophthalmic and Vision Research, and the research was approved by the institutional research board at Johns Hopkins Medical Institutions.

### Exposure to Cigarette smoke [25]

At 8 weeks of age, mice were placed into a smoking chamber for 5 hours/day, 5 days/week for 6 months. This chamber contains a smoking machine (Model TE-10, Teague Enterprises, Davis, CA) that burns 5 cigarettes (2R4F reference cigarettes (2.45 mg nicotine/cigarette; Tobacco Research Institute, University of Ky) at a time, taking 2 second duration puffs at a flow rate of 1.05 l/min, to provide a standard puff of 35 cm^3^, providing a total of 8 puffs per minute. The machine is adjusted to produce side stream (89%) and mainstream smoke (11%). The chamber atmosphere is monitored to maintain total suspended particulate at 90 mg/m^3^, and carbon monoxide at 350 ppm. Control mice were kept in a filtered air environment.

### Tissue Preparation

After mice were sacrificed and eyes were enucleated, one eye was fixed in 2.5% glutaraldehyde and 1% paraformaldehyde in 0.08 M cacodylate buffer in preparation for electron microscopy. The contralateral eye was either fixed in 2% paraformaldehyde for histochemical analysis.

### Immunohistochemical Localization of 8-Oxo-7,8-Dihydro-2′-Deoxyguanosine (8OHdG)

Cryosections (8 μm) from mice exposed to air or cigarette smoke for 6 months (n = 4 per group) were first blocked with Avidin/Biotin blocking reagent (Vector Labs, Burlingame, CA) and then a mouse on mouse blocking reagent (M.O.M.™; anti-mouse IgG blocking reagent (Vector Labs) for 1 hour at room temperature. Sections were exposed to a monoclonal anti-8-OHdG antibody (1:20; Japan Institute for the Control of Aging, Shizuoka, Japan) or mouse IgG1 for 30 minutes at room temperature. The anti-8-OHdG antibody demonstrates no cross-reactivity to 19 analogues of 8-OHdG including guanosine (G), 7-methyl-G, 6-SH-G, 8-bromo-G, dA, dC, dT, dI, dU, dG, O^6^-methyl-dG, 8-OHdA, guanine (Gua), O^6^-methyl-Gua, 8-OHGua, uric acid, Urea, creatine, creatinine. Only 8-sulfhydryl-G and 8-OHG demonstrate minimal cross-reactivity (less than 1%) [43], [44]. Sections were then incubated with Biotinylated Anti-Mouse IgG (Vector Labs) for 10 minutes at room temperature, and then with Texas Red Avidin DCS (10 μg/ml, Vector Labs). Sections were counterstained with DAPI (Vector Labs) and imaged with a confocal microscope (Zeiss 510 META confocal microscope, Carl Zeiss Micro Imaging, Inc., Thornwood, NY) at wavelengths 405/460 nm for DAPI and 633/650 nm for Texas Red. The number of nuclei positive for 8-OHdG immunostaining was counted in 5 sections per eye using the method of Dunaief et al [12]. A positive nuclei was defined by a the red color with the TMR label and distinguished from autofluorescent background by a violet colored nuclei after merging the DAPI and Texas Red label. The distribution of 8-OHdG positive cells was compared using the nonparametric, Wilcoxon exact 2-sample test.

### Ultrastructural Analysis

After fixing one eye for electron microscopic analysis as described above, the central 2×2 mm tissue temporal to the optic nerve was postfixed with 1% osmium tetroxide and dehydrated and embedded in Poly/Bed 812 resin (Polysciences, Inc., Warrington, PA). Ultrathin sections were stained with uranyl acetate and lead citrate, and examined with a JEM-100 CX electron microscope (JEOL, Tokyo, Japan) in the Wilmer Core Facility.

The average BrM thickness was determined from the thinnest and thickest measurements by a masked observer, as previously described [45]. RPE and choriocapillaris ultrastructural changes, and BrM basal deposits including location, thickness, continuity, and content were graded for severity. Each change was graded on an ordinal scale from 0 to 3 using a minimum of 50 sections by a masked observer, as previously described [31], [46]. To examine the effect of smoke on tissue ultrastructure, we used linear regression to control for clustering of results within individual mice. Linear regression analysis was performed using Stata Version 8 (Statacorp, College Station, TX).

### TUNEL assay

TUNEL assay was performed on 8 μm cryosections. Slides were dried at room temperature for 30 minutes. Tissue was permeabilized with 0.1% triton X-100 and 0.1% sodium citrate for 2 minutes on ice. TUNEL labeling was performed with an In Situ Cell Death Detection Kit, TMR (Roche, Manheim, Germany). Sections were incubated and covered with parafilm for 60 minutes at 37°C. Sections were counterstained with DAPI (Vector Labs). Positive controls were created by incubating tissue with 1 mg/ml DNAse I in 50 mM Tris-HCl, pH 7.5, 1 mM magnesium chloride, and 1 mg/ml bovine serum albumin for 10 minutes at room temperature. Coverslips were mounted with Vectashield (Vector Labs). TMR and DAPI were visualized with a confocal microscope (Zeiss 510 META confocal microscope, Carl Zeiss Micro Imaging, Inc., Thornwood, NY) at 543 nm and 455 nm, respectively. The number of TUNEL-positive cells was counted in 5 sections per eye using the method of Dunaief et al [12]. A TUNEL-positive cell was defined by a the red color with the TMR label and distinguished from autofluorescent background by a violet colored nuclei after merging the DAPI and TMR label. The distribution of TUNEL-positive cells was compared using the nonparametric, Wilcoxon exact 2-sample test.
